# Pharmacokinetics effects of chuanxiong rhizoma on warfarin in pseudo germ-free rats

**DOI:** 10.3389/fphar.2022.1022567

**Published:** 2023-01-05

**Authors:** Haigang Li, Yi Zhou, Luanfeng Liao, Hongyi Tan, Yejun Li, Zibo Li, Bilan Zhou, Meihua Bao, Binsheng He

**Affiliations:** ^1^ Hunan key laboratory of the research and development of novel pharmaceutical preparations, Changsha Medical University, Changsha, China; ^2^ Department of Pharmacy, Changsha Medical University, Changsha, China; ^3^ Academician Workstation, Changsha Medical University, Changsha, China; ^4^ Center of Clinical Pharmacology, The Third Xiangya Hospital, Central South University, Changsha, China; ^5^ Department of medical laboratory, Changsha Medical University, Changsha, China; ^6^ Department of Pharmacy, Changsha Health Vocational College, Changsha, China

**Keywords:** chuanxiong, gut microbiota, MCAO (middle cerebral artery occlusion), pharmacokinetics, UPLC-MS/MS, warfarin

## Abstract

**Aim:** In China, warfarin is usually prescribed with Chuanxiong Rhizoma for treating thromboembolism diseases. However, the reason for their combination is still being determined. The present study explored the pharmacokinetics interactions of warfarin, Chuanxiong Rhizoma, and gut microbiota in the rat model of middle cerebral artery occlusion (MCAO).

**Methods:** A total of 48 rats were randomly divided into six groups: MCAO rats orally administered warfarin (W group), pseudo germ-free MCAO rats orally administered warfarin (W-f group), MCAO rats co-administered Chuanxiong Rhizoma and warfarin (C + W group), pseudo germ-free MCAO rats co-administered Chuanxiong Rhizoma and warfarin (C + W-f group), MCAO rats co-administered warfarin and senkyunolide I (S + W group); pseudo germ-free MCAO rats co-administered warfarin and senkyunolide I (S + W-f group). After treatment, all animals’ blood and stool samples were collected at different time points. The stool samples were used for 16S rRNA sequencing analysis. Ultra-performance liquid chromatography coupled to tandem mass spectrometry (UPLC-MS/MS) method was established to quantify warfarin, internal standards, and the main bioactive components of Chuanxiong in blood samples. The main pharmacokinetics parameters of warfarin were calculated by DAS 2.1.1 software.

**Results:** The relative abundance of *Allobaculum* and *Dubosiella* in the pseudo germ-free groups (W-f, C + W-f, S + W-f) was lower than that in the other three groups (W, C + W, S + W). The relative abundance of *Lactobacillus* in the W-f group was higher than that of the W group, while the relative abundance of *Akkermansia* decreased. The relative abundance of Ruminococcaceae*_UCG-014* and Ruminococcaceae*_NK4A214_group* in the S + W-f group was lower than in the S + W group. Compared to the W group, the AUC_0-t_ and C_max_ of warfarin in the W-f group increased significantly to 51.26% and 34.58%, respectively. The AUC_0-t_ and C_max_ in the C + W group promoted 71.20% and 65.75% more than the W group. Compared to the W group, the AUC_0-t_ and C_max_ increased to 64.98% and 64.39% in the S + W group.

**Conclusion:** Chuanxiong Rhizoma and senkyunolide I (the most abundant metabolites of Chuanxiong Rhizoma aqueous extract) might affect the pharmacokinetics features of warfarin in MCAO rats through, at least partly, gut microbiota.

## Introduction

Venous thromboembolism (pulmonary embolism and deep venous thrombosis) is the third most common life-threatening cardiovascular disease. Anticoagulation is the primary treatment method for venous thromboembolism (Wilbur and Shian, 2017). As one of the most powerful oral-administered anticoagulants, warfarin is widely recommended to treat thromboembolism. As previously reported, warfarin was prescribed to ≥75% of anticoagulated patients with intracranial hemorrhage or other diseases (Chai-Adisaksopha et al., 2017; Michalcova et al., 2018). However, because of the narrow therapeutic window, the same action of warfarin that prevents blood clotting could result in bleeding. Long-term outcomes of warfarin followed by the side effect of hemorrhagic diseases, such as stroke, a well-managed warfarin treatment are indispensable.

Botanical drugs have been widely used in different diseases. Up to 80% of individuals in developing countries and 33% in developed countries took botanical drugs to treat cardiovascular diseases. Previous studies indicated that 84% of patients used botanical drugs as complementary or alternative medicine for the treatment of cardio cerebral thrombotic diseases in Singapore (Lim et al., 2018), about 26.0% and 23.5% of patients concomitantly used warfarin with botanical drugs in Hong Kong and Italy (Wong et al., 2003; Cuzzolin et al., 2007). A meta-analysis revealed that warfarin combined with botanical drugs was more effective than warfarin alone for treating thromboembolic-induced atrial fibrillation without increasing the incidence of bleeding risk (Wang et al., 2017).

Chuanxiong Rhizoma, a traditional Chinese botanical drug, has been used to treat cardiovascular diseases for centuries. According to the traditional Chinese medicine (TCM) theory (Chinese Pharmacopoeia Committee, 2020), Chuanxiong Rhizoma promotes blood circulation and alleviates blood stasis. Therefore, it is widely used in Chinese botanical formulas, such as Taohong Siwu Decoction (Wang et al., 2020b) and Buyang Huanwu Decoction (Cui et al., 2015; Chen et al., 2020), as the main component for the treatment of cardiovascular diseases with blood congestion. Moreover, warfarin was often prescribed combined with botanical formulas containing Chuanxiong Rhizoma in China to treat thromboembolism in heart diseases (Feng et al., 2015; Zhang et al., 2016). However, the underlying mechanism of their combination is still unclear.

In a previous study, we verified that oral co-administration of Chuanxiong with warfarin significantly affects the pharmacokinetics features of warfarin in healthy rats (Li et al., 2016b). As the main active metabolite of Chuanxiong, senkyunolide I was excreted into rat bile after 2 h oral administration (Li et al., 2016b). The bile-excreted drugs would be reabsorbed in the small intestine and back to the liver, sometimes with hepatic conjugation and intestinal de-conjugation (Roberts et al., 2002). This enterohepatic circulation was affected by the metabolites of gut microbiota. For example, some drugs were metabolized into hepatic phase Ⅱ metabolites, and then were de-conjugated into their prototype by β-glucuronidase, a metabolite of intestinal flora (Awolade et al., 2020). In rats, the phase II metabolic pathway of senkyunolide I mainly involve glucuronidation and methylation conjugation. The gut microbiota might participate in this enterohepatic circulation of senkyunolide I. Interestingly, the hepatic glucuronidation metabolites of warfarin could also be excreted into the bile, indicating a possible interaction between Chuanxiong and warfarin through gut microtiota.

The present study was to elucidate the effects of Chuanxiong Rhizoma on the pharmacokinetics features of warfarin in rat middle cerebral artery occlusion (MCAO) model and reveal the functions of gut microbiota in the Chuanxiong Rhizoma -warfarin interactions. This study would provide new experimental evidence to interpret the underlying mechanisms combined with botanical drug-drug interactions.

## Materials and methods

### Chemicals and reagents

Warfarin sodium (C_19_H_15_NaO_4_, CAS, 129–06-6; Lot, 18181112) was purchased from Shanghai Xinyi Jiufu Pharmaceutical Co., Ltd. (Shanghai, China). Ciprofloxacin hydrochloride (C_17_H_18_FN_3_O_3_·HCl, CAS, 93,107–08-5, Lot, 200409707) was ordered from China Meheco Topfond Pharma Co., Ltd. (Zhumadian, China). Gliclazide (C_15_H_21_N_3_O_3_S, CAS, 21,187–98-4, purity ≥98.0%; Lot, 64680050; internal standard, IS) was obtained from the ANPEL Laboratory Technologies Inc. (Shanghai, China). Senkyunolide I (C_12_H_16_O_4_, CAS, 94,596–28-8, purity ≥98%; Lot, PA0804FA13) was purchased from Target Molecule Co. (Shanghai, China). Levistilide A (C_24_H_28_O_4_, CAS, 88,182–33-6, purity ≥98.0%; Lot, AF21030107) was obtained from Chengdu Alfa Biotechnology Co., Ltd. (Chengdu, China). Ferulic acid (CAS, 1135–24-6, purity ≥99.4%; Lot, 110,773–201915) and reference substance of Chuanxiong (120,918–201910) were purchased from the National Institutes for Food and Drug Control (Beijing, China). 2,3,5-triphenyl-2H- tetrazolium chloride (TTC, C_19_H_15_ClN_4_, CAS, 298–96-4, purity ≥98.0%; Lot, EZ4567D118) was bought from BioFroxx (German, Einhausen). Acetonitrile and methanol (HPLC grade) were ordered from Merck (Darmstadt, Germany). Hexane, ethyl acetate, and ethyl ether were obtained from Sinopharm Chemical Reagent Co., Ltd. (Shanghai, China). Formic acid was purchased from Tianjing Gangfu Fine Chemical Reagent Factory (Tianjing, China). Deionized water was purified using a TKA Samrt2pure water purification system (German) with a sensitivity of 18.2 MO.

### Preparation and validation of chuanxiong rhizoma aqueous extract

Raw dried Chuanxiong Rhizoma [rhizomes of Conioselinum anthriscoides ‘Chuanxiong’ (synonym: *Ligusticum chuanxiong* S.H.Qiu, Y.Q.Zeng, K.Y.Pan, Y.C.Tang and J.M.Xu), Lot, 20190202; production date, 22 Feb 2019, Chengdu, China] was obtained from Hunan Yaoshengtang Traditional Chinese Medicine Science and Technology Co., Ltd. (Changsha, China). The botanical drug preparation of Chuanxiong Rhizoma was made according to the standard of Chinese Pharmacopoeia (2015). The specimen of Chuanxiong Rhizoma (voucher specimen no. 1908260, Chengdu, China) was identified by Professor Fang Li (Institute of Chinese Medicine, Changsha Medical University, Changsha, China). An equivalent of 100 g raw Chuanxiong Rhizoma was soaked in water at room temperature for 30 min to prepare a decoction in water (1:8 g/mL) for 1 h, followed by filtration. Subsequently, the gruffs were decocted for an hour with water (1:6 g/ml) and filtered again. The two filtrates were pooled and vacuum-dried with rotary evaporation at 50°C and concentrated to 100 ml. The final decoction was stored at 4°C for further use.

The high-performance liquid chromatography (HPLC, LC-20AT, Shimadzu, Japan) consisted of a binary solvent delivery pump, a photo-diode array (PDA) detector, and a LabSolutions data analysis system. The Chuanxiong Rhizoma aqueous extract was dissolved with methanol, diluted to an appropriate concentration, and filtered through a 0.45 µm filter membrane. The standard solutions of ferulic acid, senkyunolide I, and levistilide A were made by adding methanol to a 10 ml volumetric flask before analysis. The flow rate was adjusted to 1.0 ml/min, and the sample volume was 10 µl. The effluent was monitored at 254, 280, and 321 nm by the PDA detector. The first chromatographic separation was achieved on a WondaSil C_18_ column (4.6 mm × 250 mm, i. d., 5 μm; serial No. 9E9702–06, GL Sciences, Japan), using acetonitrile (40%) and water-glacial acetic acid (100:0.25, v/v, 60%) as mobile phase. The total run time for the separation was 50 min.

The second chromatographic separation method was achieved on a Shim-pack VP-ODS column (4.6 mm × 250 mm, i. d., 5 μm; serial No. 0062299, Shimadzu, Japan). Methanol (A) and water-glacial acetic acid (100:0.5, v/v, B) were used as mobile phase with a gradient elution: 0–15 min, 90% B linear decrease to 84% B; 15–30 min, linear decrease to 70% B; 30–35 min, linear decrease to 60% B; 35–70 min, linear decrease to 45% B. The total run time was 70 min.

### Surgical procedure

All animal procedures were approved by the Animal Ethics Committee of Changsha Medical University (No. 20190714) and performed strictly according to our institutional protocols. A total of 48 Sprague-Dawley (SD) male rats (specific pathogen-free (SPF) grade, 180–220 g) were obtained from Shanghai Laboratory Animal Center (SLAC) and housed at a temperature-controlled (22°C–26°C) and humidity-controlled (45%–75%) room, with 12 h day/night cycle with lights. All rats were fed with standard rodent chow and received tap water *ad libitum*.

As recommended by Stroke Treatment Academic Industry Roundtable (STAIR), the permanent middle cerebral artery occlusion (MCAO) is suggested as the primary animal model since it more accurately reflects and mimics the clinical stroke cases compared to the MCAO reperfusion model (McBride and Zhang, 2017). Animals were fasted for food but not water overnight before the experiments. After anesthetization by intraperitoneal injection with 10% chloral hydrate, the MCAO operation was carried out under sterile conditions. An infrared lamp was used to maintain normal body temperature. After skin preparation, rats were placed in a supine position. The right common carotid artery (CCA), the right external carotid artery, and the right internal carotid artery (ICA) were isolated and exposed carefully. A 4–0 monofilament nylon suture (2432-A1, Beijing Cinontech Co., Ltd.) coated with 1% poly-l-lysine was threaded carefully from the CCA to the ICA. The Willis circle was reached to occlude the middle cerebral artery.

The experimental rats were randomly divided into six groups (*n* = 8). Warfarin group W): MCAO rats gastric-administered 0.5 mg/kg warfarin sodium. Warfarin pseudo germ-free Group (W-f): After pre-treatment with 50 mg/kg ciprofloxacin hydrochloride for three consecutive days (twice a day), MACO rats were administered 0.5 mg/kg warfarin sodium gastrically on day 4. Chuanxiong Rhizoma combined warfarin group (C + W): MCAO rats were administered Chuanxiong Rhizoma and warfarin sodium at doses of 10 g/kg and 0.5 mg/kg, respectively, gastrically. Chuanxiong Rhizoma combined warfarin under pseudo germ-free condition group (C + W-f): After pre-treatment with ciprofloxacin hydrochloride, MCAO rats were administered Chuanxiong Rhizoma and warfarin sodium at doses of 10 g/kg and 0.5 mg/kg, respectively, gastrically. Senkyunolide I combined warfarin group (S + W): MCAO rats were administered senkyunolide I and warfarin sodium at doses of 75 mg/kg and 0.5 mg/kg, respectively, gastrically. Senkyunolide I combined warfarin under pseudo germ-free group (S + W-f): After pre-treatment with ciprofloxacin hydrochloride, MCAO rats were gastrically administered senkyunolide I and warfarin sodium at doses of 75 mg/kg and 0.5 mg/kg, respectively. This study’s dose of Chuanxiong Rhizoma was calculated according to the dosage used clinically. In the traditional formula, the maximum conventional clinical dosage of Chuanxiong Rhizoma is 30 g. Considering the simple recipe of Chuanxiong Rhizoma, its dosage was up-regulated in the present protocol, and the content of senkyunolide I in the aqueous extract of Chuanxiong Rhizoma was about 0.75 mg/g (Yan et al., 2005).

### Sample collection and preparation

The blood samples of rats were collected into heparinized tubes at 0 min, 5 min, 15 min, 30 min, 60 min, 2 h, 4 h, 8 h, 12 h, and 24 h after drug treatment. Before collecting the blood samples, all animals were given 5 ml of Ringer’s solution subcutaneously to avoid the excessive loss of body liquids. After the blood samples were collected, the caecum stools were collected and stored at −80°C for further analysis.

The supernatants were collected by centrifugation of blood samples at 3,000 rpm for 15 min and stored at −80°C until analysis. An aliquot of 100 μl of rat plasma, 10 μl of internal standard (gliclazide solution), and 50 μl of 20% formic acid was added to 840 μl of methanol. The above mixture was vortex-mixed for 3 min, hyper acoustic mixed for 5 min, and centrifuged at 3,000 rpm for 10 min. Under a gentle stream of nitrogen, the supernatants were evaporated to dryness at room temperature. The dried samples were reconstituted with a 100 μl mixture of acetonitrile and water (50:50), clarified by centrifugation at 15,000 rpm for 10 min, and filtered through the 0.22 μm membrane. A volume of 2 μl of the processed sample was injected for the analysis.

### TTC staining

After anesthetized by chloral hydrate, the rat brain was collected on ice and stored at −20°C for 30 min. Thin coronal slices (2 mm) of the brains were stained by 1% TTC dye solution (in PBS, pH 7.4) for 20 min at 37°C in the dark. The stained slices were fixed with 4% paraformaldehyde for 24 h, and then the images of these slices were analyzed.

### UPLC-MS/MS analysis

The UPLC-MS/MS system was used to determine the levels of warfarin, levistilide A, and senkyunolide I in blood samples. The Acquity™ UPLC system (Waters Corporation, Milford, MA, United States) was composed of a column oven (set at 35°C), a binary solvent delivery manager, and an autosampler (set at 10°C). The chromatographic separation was achieved on a Waters Acquity BEH C_18_ column (2.1 mm × 100 mm I.D., 1.7 μm, Waters, Wexford, Ireland). A mobile phase containing A (aqueous buffer containing 0.1% formic acid) and B (acetonitrile) was pumped at 0.3 ml/min. The following linear gradient elution was applied to the analyte: 0–2.0 min, 10% B; 2.0–5.0 min, 10%–95% B; 5.0–8.0 min, holding at 95% B; between 8.0 and 10.0 min, decrease to 10% B (the initial conditions) for equilibration of the column.

The typical injection volume was 2 μl. The detection system and a tandem quadrupole mass spectrometer (Waters Corporation, Manchester, United Kingdom) were operated using electrospray ionization with the capillary voltage set at 2.5 kV, the desolvation temperature was fixed at 365°C, and the source temperature was set at 110°C in the negative ion mode. Nitrogen was used for the cone gas flow (50 L/h) and desolvation gas flow (650 L/h). Argon was used as the collision gas at a flow rate of 0.2 ml/min for collision-induced dissociation. MassLynx 4.1 software program (Waters Corporation) was used for data acquisition and processing. The multiple-reaction monitoring (MRM) mode was selected for the quantitation of IS and the main bioactive components of Chuanxiong. The data were collected and analyzed using the DAS 2.1.1 software.

### Microbial analysis of rat stool

After the final blood sample was taken, the caecal content of all rats was collected for gut microbiota analyses. Briefly: (Ⅰ) genomic DNA was extracted; (Ⅱ) the 16S rRNA V3-V4 region was amplified using the 338F (5′-ACT​CCT​ACG​GGA​GGC​A GCA-3′) and 806R (5′-GGACTACHVGGGTWTCTAAT-3′) primers; (Ⅲ) PCR product purification, quantification, and qualification; (Ⅳ) library preparation and sequencing were performed on a MiSeq platform (Illumina, San Diego, United States).

The 16S rRNA sequencing data were quality-filtered using fast length adjustment of short reads (FLASH, v1.2.11). The operational taxonomic units (OTUs) were identified at a 97% sequence similarity cut-off using USEARCH (v7.0.1090) and aligned using Silva (Release128 http://www.arb-silva.de). Moreover, the ribosomal database project (RDP, v2.2) classifier was used to classify the OTUs at a given taxonomic rank.

### Statistical analysis

Statistical evaluation was analyzed by SAS software (Cary, North Carolina). Data were presented as the means with standard deviation (SD). The differences between the quantitative groups with normal distribution were evaluated with the Student’s t-test. The Mann-Whitney test was used for abnormally distributed variables. Differences were considered statistically significant for *p* < 0.05.

## Results

### Validation of chuanxiong rhizoma aqueous extract

The HPLC analytical results are shown in Supplementary Figure S1. The first chromatographic separation method (with WondaSil C_18_ column) was suitable for the detection ferulic acid (R_t_ = 10.650 min) and senkyunolide I (R_t_ = 18.473 min) in the aqueous extract of Chuanxiong Rhizoma (Fig. S1-A). The second chromatographic separation method (with Shim-pack VP-ODS column) displayed a low resolution in the separation of ferulic acid and senkyunolide I. The peak of levistilide A (R_t_ = 62.585 min) was detected at wavelength 254 nm (Supplementary Figure S1-B).

### Establishment of LC-MS/MS method

**FIGURE 1:**
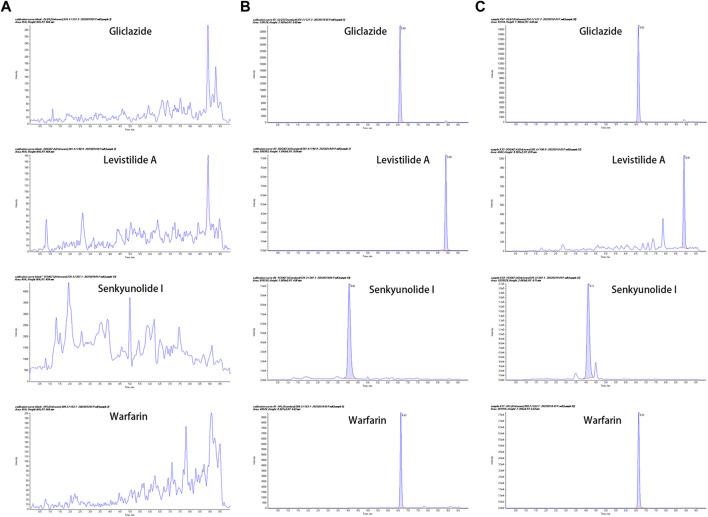
The representative MRM chromatograms **(A)** Blank plasma samples from MCAO rats; **(B)** Blank plasma samples spiked with gliclazide (IS), levistilide A, senkyunolide I, and warfarin; **(C)** Plasma from MCAO rats after co-administration of warfarin and Chuanxiong Rhizoma. (From the top to the bottom: gliclazide, levistilide A, senkyunolide I, and warfarin).

The established LC-MS/MS method displayed good specificity, with no interference at the retention times of warfarin, levistilide A, and senkyunolide I. The typical MRM chromatograms are shown in Figure 1. The linear regression equation of warfarin was *y* = 0.0025*x +* 0.0512 (*r* ^*2*^ = 0.999250), where *x* referred to the warfarin concentration (ng/ml), *y* indicated the ratio of the peak area of warfarin to that of internal standard, and *r* was the correlation coefficient of the equation. The linearity range was 5–10′000 ng/ml, and the lower limit of quantification (LLOQ) was 5 ng/ml. All the results were within the accepted variable limits.

### TTC result of MCAO model

**FIGURE 2:**
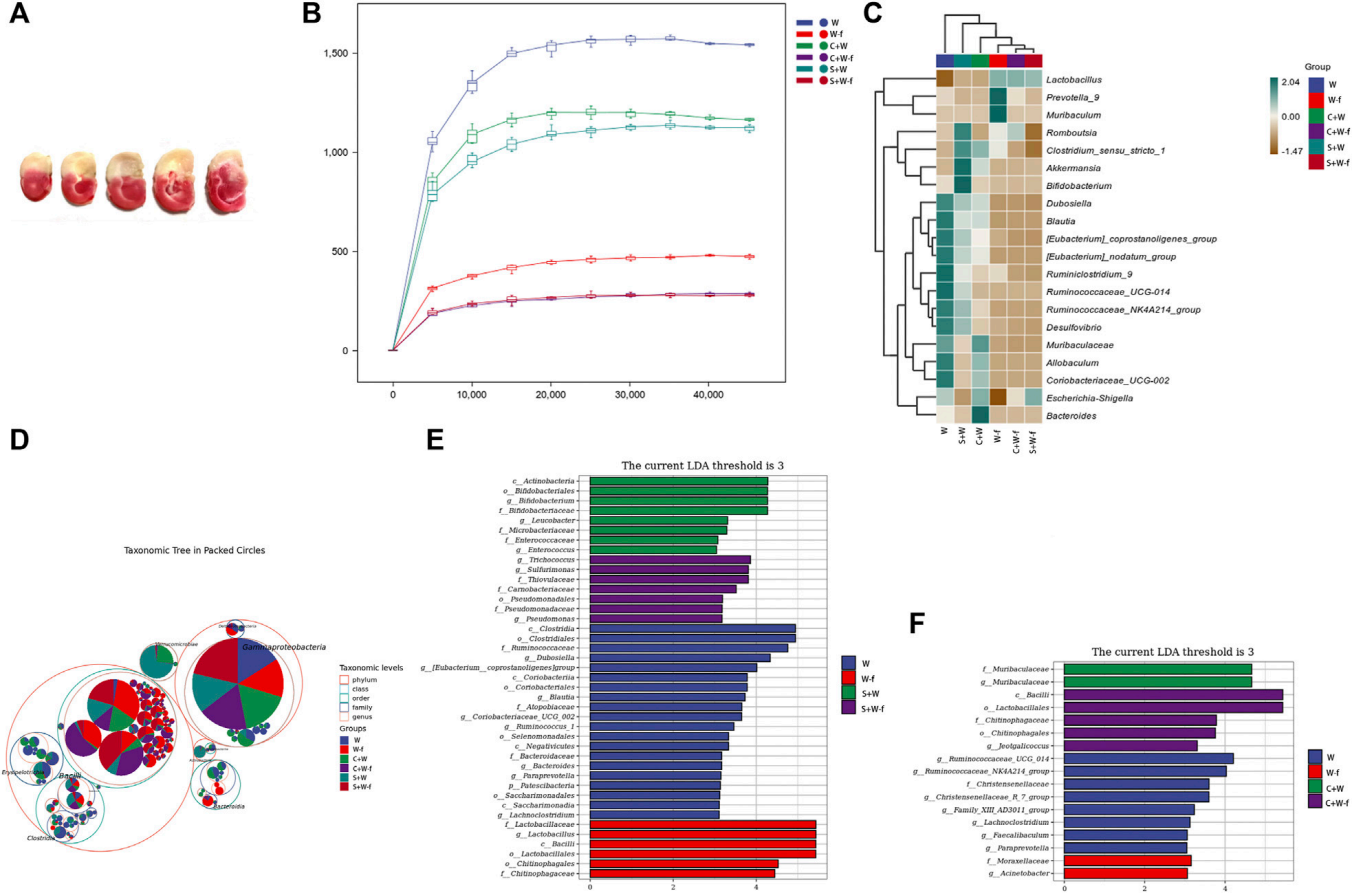
The TTC staining of the brain of MCAO rats and the results of 16S rRNA sequencing of the gut microbiota **(A)** The TTC staining of MCAO rats; **(B)** The rarefaction curves of Chao1 index; **(C)** Heatmap of the species compositions; **(D)** Taxonomic tree in packed circles; **(E, F)** The linear discriminant analysis (LDA) effect size algorithm (LEfSe) analysis of the gut microbiota. W: MCAO rats with oral administration of warfarin; W-f: pseudo germ-free MCAO rats with oral administration of warfarin; C + W: MCAO rats with oral co-administration of warfarin and Chuanxiong Rhizoma; C + W-f: pseudo germ-free MCAO rats with oral co-administration of warfarin and Chuanxiong Rhizoma. S + W: MCAO rats with oral co-administration of warfarin and senkyunolide I; S + W-f: pseudo germ-free MCAO rats with oral co-administration of warfarin and senkyunolide I.

TTC staining (Figures 2–A) showed histological changes in cerebral tissues. The occluded lateral brain was white in color, indicating infarction, while the non-operation lateral brain displayed uniform red, indicating no infarction. The results of TTC staining indicated that the rat MCAO model was established successfully.

### Relative abundance of gut microbiota

To further investigate the mechanisms underlying the alteration of Chuanxiong-induced warfarin pharmacokinetics, we measured the changes in gut microbiota in MCAO rats using 16S rRNA high-throughput sequencing. The different gut microbiota composition was presented in rarefaction curves of the Chao1 index (Figures 2–B). The OTUs of three pseudo germ-free groups (including W-f, C + W-f, and S + W-f) were lower than those of the other three groups. This founding was further supported by the heat map of species composition (Figures 2–C); the diversity in the bacteria species composition of the three pseudo germ-free groups presented some homogeneity. Chuanxiong Rhizoma and senkyunolide I decreased the diversity of bacteria compared to the W-f and W groups. It also showed obvious differences in the diversity of bacteria composition among the groups in the taxonomic tree in packed circles (Figures 2–D). We also utilized the linear discriminant analysis (LDA) effect size algorithm (LEfSe) analysis to evaluate the classification differences in the gut microbiota among the six groups (Figures 2–E,F).

**FIGURE 3:**
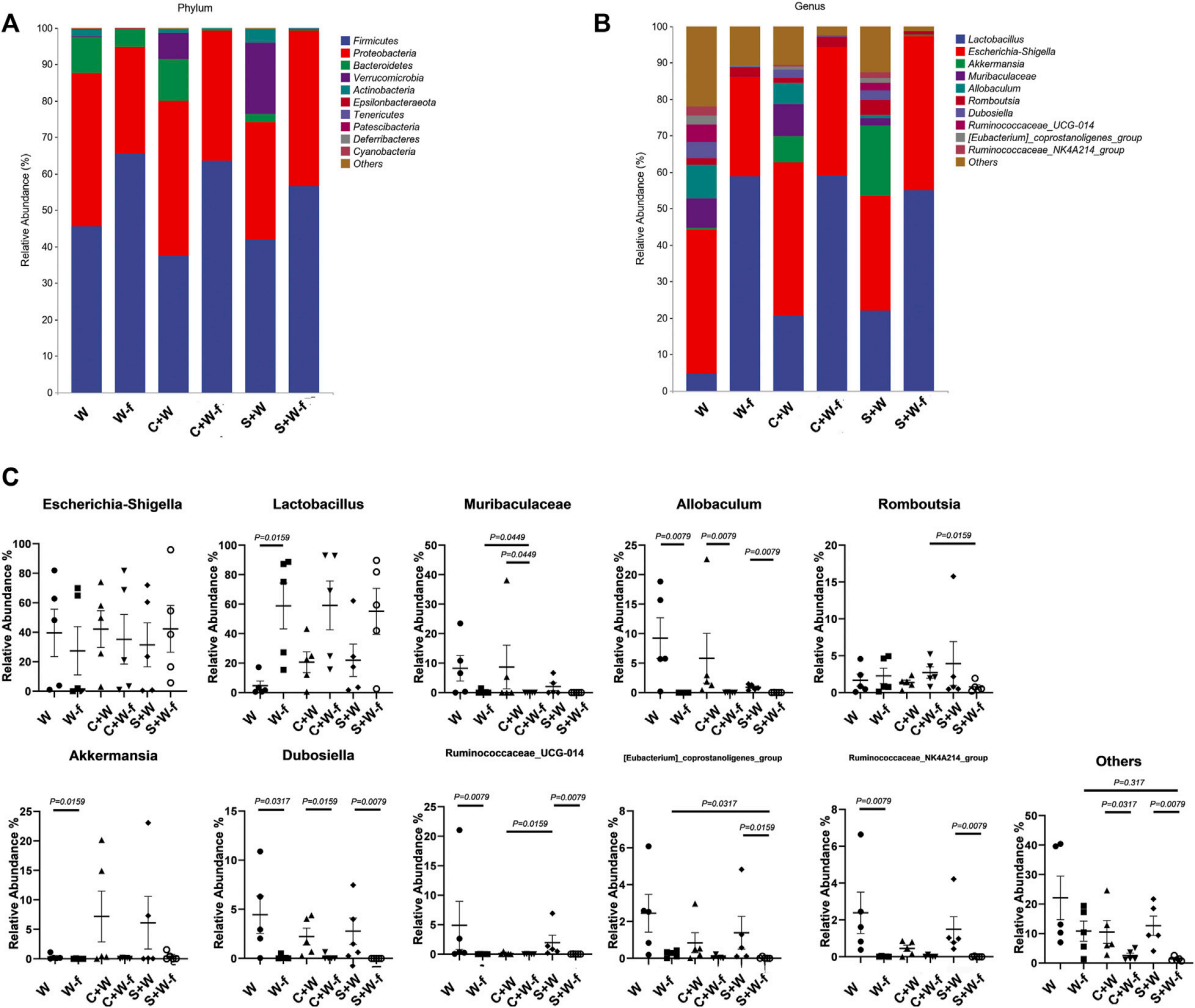
The relative abundance of gut microbiota compositions **(A)** At the phylum level and **(B)** genus level; **(C)** significant bacteria on the relative abundance at the genus level. W: MCAO rats with oral administration of warfarin; W-f: pseudo germ-free MCAO rats with oral administration of warfarin; C + W: MCAO rats with oral co-administration of warfarin and Chuanxiong Rhizoma; C + W-f: pseudo germ-free MCAO rats with oral co-administration of warfarin and Chuanxiong Rhizoma. S + W: MCAO rats with oral co-administration of warfarin and senkyunolide I; S + W-f: pseudo germ-free MCAO rats with oral co-administration of warfarin and senkyunolide I.

The phylum and genus levels of the gut microbiota structures in the six groups are displayed in Figure 3. Firmicutes were the highest abundant bacteria in the pseudo germ-free groups (W-f, C + W-f, and S + W-f); they shared 65.7%, 63.6%, and 56.6% of the total bacteria, respectively. The average ratio of *Firmicutes* in the W, C + W, and S + W groups was 45.6%, 37.5%, and 41.9% of the total bacteria, respectively.

We also found obvious differences in the relative abundance in nine of the top ten genera among the six groups. The relative abundance of *Allobaculum* and *Dubosiella* of the pseudo germ-free groups was lower (*p <* 0.050) than that in the other three groups (Figures 3–C). The relative abundance of *Lactobacillus* in the W-f group was higher than that of the W group due to the pre-treatment antibiotic (*p =* 0.0159). However, the relative abundance of *Akkermansia* decreased (*p =* 0.0159). The differences between the S + W and S + W-f groups were more than those between the C + W and C + W-f groups.

The ciprofloxacin-induced pseudo germ-free condition affected the bacteria composition obviously. We found that the relative abundance of Ruminococcaceae*_UCG-014* and that of Ruminococcaceae*_NK4A214_group* in the S + W-f group were remarkably decreased compared to the S + W group (*p* = 0.0079). A similar result was observed between the W and W-f groups.

The combination of Chuangxiong or senkyunolide I affected the relative abundance of bacteria. The average relative abundance of *Lactobacillus* in the W group was only 4.70% (median 1.65%), which increased to 20.65% (median 22.55%) in the C + W group, and 17.44% (median 21.92%) in the S + W group. The average relative abundance of *Allobaculum* in the W group was 9.24% (median 5.76%), which decreased to 5.83% (median 1.87%) in the C + W group and 0.79% (median 0.90%) in the S + W group. Nevertheless, there was no significant difference in gut bacteria composition among the W, C + W, and S + W groups.

In ciprofloxacin-induced germ-free condition, Chuangxiong Rhizoma or senkyunolide I affected the less relatively abundant genus, such as Muribaculaceae and *[Eubacterium]_coprostanoligenes_group*. The relative abundance of Muribaculaceae in the C + W-f group was higher, while the relative abundance of *[Eubacterium]_coprostanoligenes_group* in the S + W-f group was lower than that of the W-f group (*p* < 0.05).

### Difference in pharmacokinetics features of warfarin after co-administered with chuanxiong rhizoma

**FIGURE 4:**
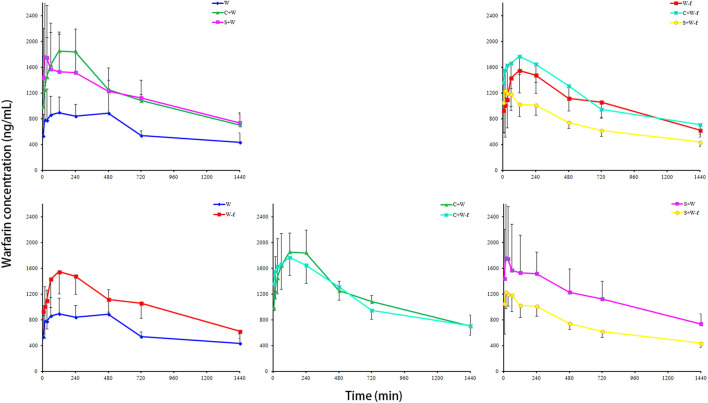
The pharmacokinetics effects of Chuanxiong Rhizoma on warfarin in MCAO rats (n = 8). W: MCAO rats with oral administration of warfarin; W-f: pseudo germ-free MCAO rats with oral administration of warfarin; C + W: MCAO rats with oral co-administration of warfarin and Chuanxiong Rhizoma; C + W-f: pseudo germ-free MCAO rats with oral co-administration of warfarin and Chuanxiong Rhizoma. S + W: MCAO rats with oral co-administration of warfarin and senkyunolide I; S + W-f: pseudo germ-free MCAO rats with oral co-administration of warfarin and senkyunolide I.

Figure 4 shows the concentration-time curves for plasma warfarin in the six groups (n = 8). The pharmacokinetics parameters (Table 1) of warfarin were calculated using the non-compartmental modeling with the DAS 2.1.1 software. Table 1 indicated significant differences in the pharmacokinetics parameters of warfarin between the W and W-f groups. Compared to the W group, the AUC_0-t_ and C_max_ of warfarin in the W-f group increased significantly by 51.26% and 34.58%, respectively. The T_max_ was 219 min earlier than the W group (*p* < 0.05). While compared to the S + W group, the AUC_0-t_ and C_max_ of warfarin in the S + W-f group decreased markedly by 38.45% and 36.45%, respectively. No obvious differences were observed between the C + W and C + W-f groups.

**TABLE 1:** The pharmacokinetics parameters of warfarin (mean ± SD, *n* = 8).

Group	AUC_(0-t)_ (ng/mL·min)	MRT_(0-t)_ (min)	t_1/2z_ (min)	T_max_ (min)	C_max_ (ng/ml)
W	1′002′828.4 ± 120′517.3	597.2 ± 97.0	1′108.1 ± 622.5	435.0 ± 588.8	1′281.5 ± 477.6
W-f	1′516′888.9 ± 158′281.2^**^	597.3 ± 54.3	1′165.8 ± 450.1	216.0 ± 283.3^a^	1′724.6 ± 357.1^**^
C + W	1′716′848.8 ± 67′833.4^**^	573.5 ± 65.5	1′017.8 ± 590.9	138.0 ± 98.6^**^	2′124.1 ± 318.7^**^
C + W-f	1′622′903.9 ± 192′269.7	580.1 ± 23.8	998.0 ± 149.9	105.0 ± 90.0	1′968.4 ± 137.0
S + W	1′654′515.6 ± 394′360.9^**^	607.0 ± 43.4	1′342.6 ± 514.2	157.0 ± 314.9^**^	2′106.7 ± 617.1^**^
S + W-f	1′018′336.1 ± 118′840.5^##andand$$^	580.0 ± 27.2	1′088.7 ± 952.2	70.0 ± 97.3	1′338.8 ± 272.7^##andand$$^

^a^ , compared to the W group, *p* < 0.05, ** compared to the W group *p* < 0.01; ##, compared to the S + W group, *p* < 0.01; andand, compared to the W-f group *p* < 0.01; $$, compared to the C + W-f group *p* < 0.01.

W: MCAO, rats with oral administration of warfarin; W-f: pseudo germ-free MCAO, rats with oral administration of warfarin; C + W: MCAO, rats with oral co-administration of warfarin and Chuanxiong Rhizoma; C + W-f: pseudo germ-free MCAO, rats with oral co-administration of warfarin and Chuanxiong Rhizoma; S + W: MCAO, rats with oral co-administration of warfarin and senkyunolide I; S + W-f: pseudo germ-free MCAO, rats with oral co-administration of warfarin and senkyunolide I.

Significant differences were detected in the pharmacokinetics parameters of warfarin between the C + W and W groups. Compared to the W group, the AUC_0-t_ and C_max_ of warfarin in the C + W group increased by 71.2% and 65.75%, respectively, and the T_max_ was earlier 297 min than that of the W group (*p* < 0.05). At the same time, co-administration of senkyunolide I (S + W group) markedly promoted the AUC_0-t_ and C_max_ of warfarin at 64.98% and 64.39%, respectively, and the T_max_ was 278 min forward than that of the W group (*p* < 0.05). Compared to the W-f group, the AUC_0-t_ of the S + W-f group decreased 32.87% (*p* < 0.01). Compared to the C + W-f group, the AUC_0-t_ and C_max_ of warfarin in the S + W-f group decreased 37.25% and 31.98%, respectively. No obvious differences were observed between W-f and C + W-f groups.

## Discussion

The severe adverse events of warfarin are a major concern in clinics. Although we explained this phenomenon based on genetic variants or other methods, >33% of individual sensitivity variation had not been explained (Wang et al., 2020a). A previous study showed that the *Escherichia-Shigella* genus was significantly richer in low warfarin responder patients, and the *Enterococcus* genus was abundant in high responders (Wang et al., 2020a). This phenomenon prompted us to hypothesize that gut microbiota might affect the patient’s sensitivity to warfarin.

In order to investigate the effect of gut microbiota on the botanical drug-drug interaction of Chuanxiong Rhizoma and warfarin, we used the broad-spectrum antibiotic ciprofloxacin as a tool drug to simulate the germ-free state. We found that the ciprofloxacin-induced germ-free condition affected the constituents of gut microbiota and warfarin disposition in a rat model of MCAO. Compared to the W group, the relative abundance of the *Lactobacillus* genus in the W-f group increased significantly, but the relative abundance of *Allobaculum, Akkermansia, Dubosiella* *,* Ruminococcaceae*_UCG-014,* and Ruminococcaceae*_NK4A214_group* decreased markedly. Accompanied by the changes in gut microbiota, the AUC_0-t_ and C_max_ of warfarin increased by 51.26% and 34.58%, respectively. These results indicated that the relative abundance and composition of the gut microbiota might be involved in the disposition of warfarin in MCAO rats.

The plasma concentration-time curves of warfarin displayed double peaks in both the W and W-f groups. Pre-treatment of ciprofloxacin did not affect the occurrence of the second peak of warfarin and only promoted the first peak (C_max_) significantly. Briefly, broad-spectrum antibiotic ciprofloxacin-induced germ-free state affected the bioavailability of warfarin significantly. The gut microbiota considers playing critical roles in the drug disposition processes (Zhang et al., 2018). The current results indicated a vital role of gut microbiota, such as *Lactobacillus, Allobaculum*, and *Dubosiella,* in the Chuanxiong Rhizoma-warfarin interactions. Within a certain range, increasing the relative abundance of *Lactobacillus* and decreasing that of *Allobaculum* and *Dubosiella* might promote the bioavailability of warfarin in the rats of the MCAO model.

As reported previously, Chuanxiong Rhizoma aqueous extract increased the AUC_0–t_, and C_max_ of warfarin to 2.35- and 2.42-fold of normal rats control (Li et al., 2016b). However, in MACO rat plasma, AUC_0-t_ and C_max_ of warfarin promoted only 71.20% and 65.75%, respectively. Altered constituents might interpret this phenomenon and the relative abundance of gut microbiota induced by the MCAO operation. Importantly, the MCAO operation markedly alters the gut microbiota composition, including an overgrowth of the non-benificial *Bacteroidetes* phylum (including *Bactreoides* and *Escherichia-Shigella* genus) to some extent and the decline in beneficial bacteria species (Singh et al., 2016; Chen et al., 2018).

Chuanxiong Rhizoma contains >100 types of pharmacologically active metabolites, such as senkyu nolide I, senkyunolide A, levistilide A, ferulic acid, ligustilide, and tetramethylpyrazine. Among these metabolites, ferulic acid modulated the gut microbiota, specifically decreasing the relative abundance of *Ileibacterium* and increasing *Lactobacillus* in the mice model of atherosclerosis (Gu et al., 2021). A recent study reported that Chuanxiong Rhizoma remodeled the gut microbiota dysbiosis induced by MCAO (Chen et al., 2019). Both warfarin and senkyunolide I effectuate enterohepatic circulation, a process regulated by gut microbiota (Li et al., 2016a; Li et al., 2016b; Li et al., 2018). This study showed that the ciprofloxacin-induced germ-free condition and co-administration of Chuanxiong Rhizoma aqueous extracts decreased the relative abundance of *Dubosiella*. *Dubosiella* showed a negative correlation with the expression of the pro-inflammatory genes in mice models of colitis (Wan et al., 2021), and inhibition of gut inflammation might promote the absorption of intestinal-absorbed drugs. The abundance of *Lactobacillus* benefits the intestinal barrier by enhancing the tight gut epithelial junctions (Miyauchi et al., 2009), and the relatively intact intestinal function should be beneficial for drug absorption. Typically, our studies discovered that the gut microbiota significantly affects the pharmacokinetics of warfarin. Gut microbiota might mediate this influence of Chuanxiong Rhizoma on the pharmacokinetics alteration of warfarin in the rat model of MCAO. Thus, additional studies are required to verify the mechanism underlying this phenomenon.

Based on the results of pharmacokinetics, Chuanxiong Rhizoma, senkyunolide I, and pre-treatment of ciprofloxacin markedly promoted the bioavailability of warfarin in the rat model of MCAO. The strongest influence on warfarin pharmacokinetics was Chuanxiong Rhizoma; with co-administration of Chuanxiong Rhizoma, the ciprofloxacin-induced germ-free condition did not affect the pharmacokinetics of warfarin significantly. Non-etheless, with or without co-administration of senkyunolide I, ciprofloxacin pre-treatment produced an opposite tendency on the pharmacokinetics of warfarin. Moreover, there were fewer OTUs of gut microbiota in the S + W-f group than in the W-f group. Compared to senkyunolide I, Chuanxiong Rhizoma aqueous extract (with multiple active metabolites) had more compatibility in gut microbiota regulation. The regulation of bacterial composition in a suitable range promoted the availability of warfarin in the rat model of MCAO.

Typically, gut microbiota might affect the pharmacokinetics of warfarin. Chuanxiong Rhizoma or senkyunolide I could exert similar effects on warfarin, indicating that similar changes existed in regulating gut microbiota composition. They altered the pharmacokinetics feature of warfarin through, at least partly, gut microbiota, such as *Lactobacillus*, *Allobaculum*, and *Dubosiella.* Our study provided more theoretical reasons for the combination of Chuanxiong Rhizoma and warfarin from the visual of gut microbiota.

## Data Availability

The datasets presented in this study can be found in online repositories. The names of the repository/repositories and accession number(s) can be found below: NCBI BioProject, PRJNA873289.

## Glossary

AUC: area under the curve of concentration-time
CCA: common carotid artery
C_max_: maximum plasm concentration
C + W: MCAO rats co-administered Chuanxiong Rhizoma and warfarin
C + W-f: pseudo germ-free MCAO rats co-administered Chuanxiong Rhizoma and warfarin
HPLC: high performance liquid chromatography
ICA: internal carotid artery
IS: internal standard
LDA: linear discriminant analysis
LEfSe: linear discriminant analysis effect size algorithm
LLOQ: lower limit of quantification
MCAO: middle cerebral artery occlusion
MRM: multiple-reaction monitoring
MRT: mean residence time
OTUs: Operational taxonomic units
PDA: photo-diode array
SD: standard deviation
R_t_: retention time
SLAC: Shanghai laboratory animal center
S + W: MCAO rats co-administered warfarin and senkyunolide I
S + W-f: pseudo germ-free MCAO rats co-administered warfarin and senkyunolide I
t_1/2_: half-life
TCM: traditional Chinese medicine
TLC: thin layer chromatography
T_max_: time to reach the maximum concentration
TTC: 2,3,5-triphenyl-2H-tetrazolium chloride
UPLC-MS/MS: ultra-performance liquid chromatography coupled to tandem mass spectrometry
UV: ultraviolet
W: MCAO rats administered warfarin
W-f: pseudo germ-free MCAO rats administered warfarin.
