# Supplementary figures and images for: Pharmacokinetics effects of chuanxiong rhizoma on warfarin in pseudo germ-free rats
Source: Front Pharmacol. 2023 Jan 5;13:1022567. doi: 10.3389/fphar.2022.1022567 (PMC9849362; doi:10.3389/fphar.2022.1022567)

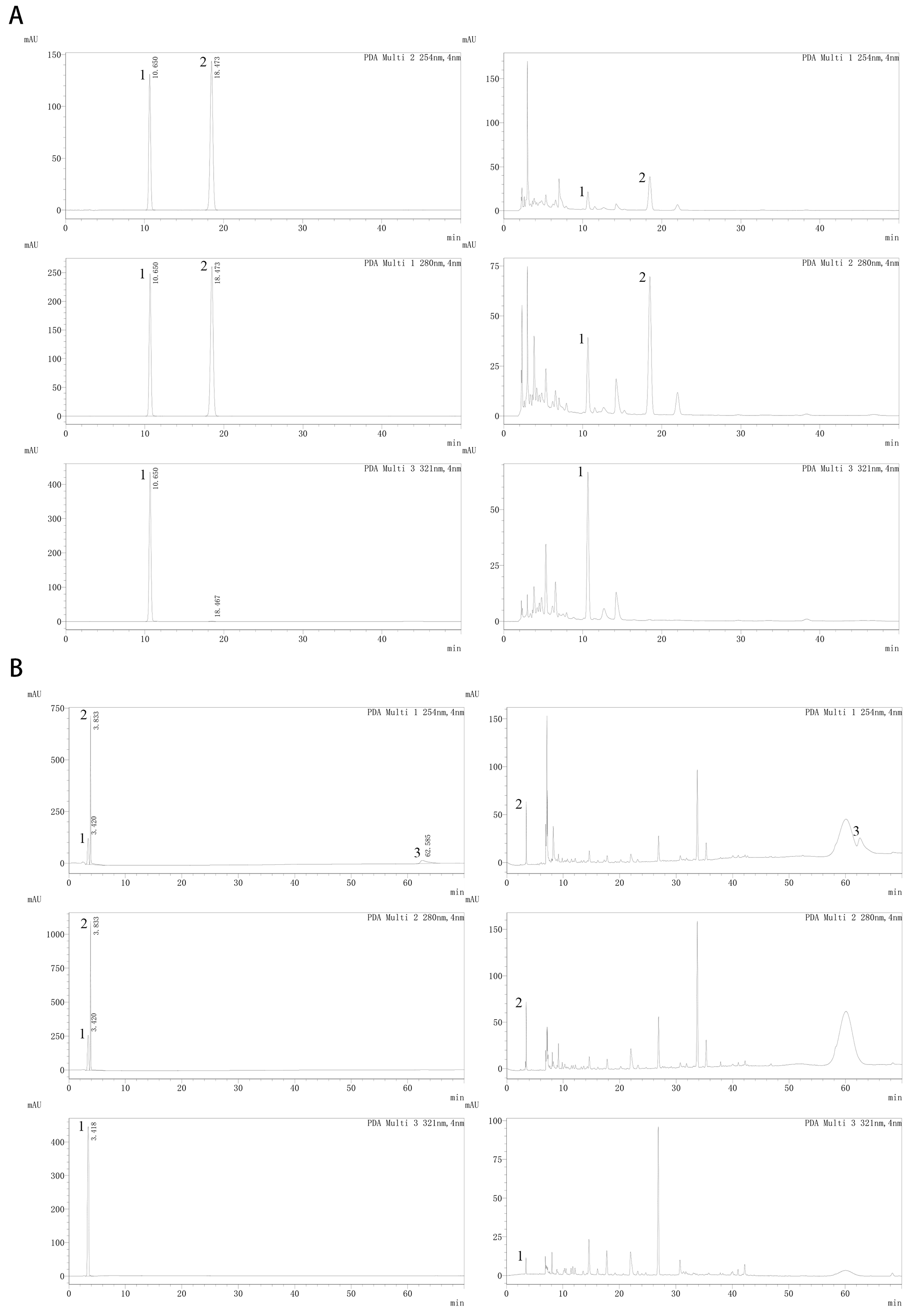

Supplement: Supplementary file 1 [file Image1.TIF]
