# Supplementary material for: Pharmacokinetics effects of chuanxiong rhizoma on warfarin in pseudo germ-free rats
Source: Front Pharmacol. 2023 Jan 5;13:1022567. doi: 10.3389/fphar.2022.1022567 (PMC9849362; doi:10.3389/fphar.2022.1022567)
Supplement: Supplementary file 2 [file DataSheet1.PDF]

Supplementary Materials

Pharmacokinetics effects of Chuanxiong Rhizoma on warfarin in pseudo germ-free rats

Haigang Li [1, 2, 3 *], Yi Zhou [1, 2, 3], Luanfeng Liao [2], Hongyi Tan [4], Yejun Li [4],
Zibo Li [5], Bilan Zhou [6], Meihua Bao [1, 3 *], Binsheng He [1,3 *]

[1] Hunan key laboratory of the research and development of novel pharmaceutical preparations, Changsha Medical University, Changsha, 410219, PR China

[2] Department of pharmacy, Changsha Medical University, Changsha, 410219, PR China

[3] Academician Workstation, Changsha Medical University, Changsha, 410219, PR China

[4] Center of Clinical Pharmacology, The Third Xiangya Hospital, Central South University, Changsha, 410013, PR China

[5] Department of medical laboratory, Changsha Medical University, Changsha, 410219, PR China

[6] Changsha Health Vocational College, Changsha, 410600, PR China

*** Corresponding author:**

Haigang Li, Changsha Medical University, 1501 Leifeng Road, Changsha, 410219, PR China
Tel: +86 731 88602635; Fax: +86 731 88602635
E-mail address: lihaigang2020@126.com

Meihua Bao, Changsha Medical University, 1501 Leifeng Road, Changsha, 410219, PR China
Tel: +86 731 88602859; Fax: +86 731 88602859
E-mail address: mhbao78@163.com

Binsheng He, Changsha Medical University, 1501 Leifeng Road, Changsha, 410219, PR China
Tel: +86 731 88602666; Fax: +86 731 88602666

E-mail address: hbscsmu@163.com

This file contains 2 Tables and 1 Figure.

I, Stability of UPLC-MS/MS method

Six samples per level at three quality control concentrations (low, middle, high) on the same day was analyzed to assess the intra-day accuracy and precision, and on three consecutive days, the three quality control samples were analyzed for evaluating the inter-day accuracy and precision.

Recovery was performed at low, middle and high quality control levels (n=6) by comparing the response of analytes for precipitated quality control samples with standards spiked after precipitation at the same concentration level. Matrix effect was investigated with different blank matrices from individual donors.

Stability was tested by using triplicates of quality control samples stored under different conditions. The autosampler stability was evaluated by analyzing extracted quality control samples kept under autosampler condition (4 ℃) for 24 h. Room temperature stability was assessed by using untreated quality control samples kept at room temperature for 24 h and long-term stability was assessed by using samples stored at −80 °C for 35 days. The freeze-thaw stability of the analytes was determined over three freeze-thaw cycles. With each cycle, the samples were frozen and stored at −20 ℃ for 24 h and then thawed at room temperature. The stability of stock solutions and working solutions of analytes and internal standard were evaluated by comparing the peak area of the stock solutions and working solutions kept at 4 ℃ for 35 days with that of the freshly prepared solutions.

Table S1. Results of precision, accuracy, matrix effect and extraction recovery of warfarin, senkyunolide I and levistilide A

Target analytes	Nominal level (nmol/L)	Precision RSD%		Accuracy %	Matrix effect %	Recovery/%
		Intra-day	Inter-day			
warfarin	5.14	3.51	4.54	98.01	102.46	99.62
	250.27	3.36	4.31	101.52	103.39	98.29
	2500.00	3.58	3.68	103.03	103.28	101.35
senkyunolide I	8.29	4.92	6.45	94.58	108.21	105.34
	81.21	5.36	5.85	93.09	107.86	104.93
	400.00	4.75	5.74	98.82	109.95	102.38
levistilide A	8.16	7.89	9.76	94.41	106.13	102.87
	80.24	7.73	8.68	95.85	107.78	105.43
	400.00	6.41	7.54	103.01	104.71	107.35

Table S2. Results of stability tests for warfarin, senkyunolide I and levistilide A.

Target analytes	Nominal level (nmol/L)	Room temperature (24 h, %)	4 ℃ Autosampler (24 h, %)	-20℃ freeze-thaw (3 times, %)	-80℃ Refrigerator 35 days, %
warfarin	5.14	97.86	101.23	101.65	103.94
	250.27	99.45	103.73	101.16	103.58
	2500.00	96.81	97.95	99.58	103.17
senkyunolide I	8.29	94.73	105.1	105.57	104.65
	81.21	96.88	104.13	102.67	105.28
	400.00	96.69	103.85	101.19	104.73
levistilide A	8.16	91.97	95.49	102.81	103.29
	80.24	98.83	93.54	107.22	107.25
	400.00	98.85	95.64	108.14	106.91

Ⅱ, **Validation of Chuanxiong Rhizoma aqueous extract**

The high-performance liquid chromatography (HPLC, LC-20AT, Shimadzu, Japan) consisted of a binary solvent delivery pump, a photo-diode array (PDA) detector, and a LabSolutions data analysis system. The Chuanxiong Rhizoma aqueous extract was dissolved with methanol, diluted to an appropriate concentration, and filtered through a 0.45 μm filter membrane. The standard solutions of ferulic acid, senkyunolide I, and levistilide A were made by adding methanol to a 10 mL volumetric flask before analysis. The flow rate was adjusted to 1.0 mL/min, and the sample volume was 10 μL. The effluent was monitored at 254, 280 and 321 nm by the PDA detector. The first chromatographic separation method was achieved on a WondaSil C_{18} column (4.6 mm × 250 mm, i.d., 5 μm; serial No. 9E9702-06, GL Sciences, Japan). Acetonitrile (40%) and water-glacial acetic acid (100:0.25, v/v, 60%) were used as mobile phase. Total run time was 50 min.

The second chromatographic separation method was achieved on a Shim-pack VP-ODS column (4.6 mm × 250 mm, i.d., 5 μm; serial No. 0062299, Shimadzu, Japan). Methanol (A) and water-glacial acetic acid (100:0.5, v/v, B) were used as mobile phase with a gradient elution: 0-15 min, 90% B linear decrease to 84% B; 15-30 min, linear decrease to 70% B; 30-35min, linear decrease to 60% B; 35-70 min, linear decrease to 45% B. Total run time was 70 min.

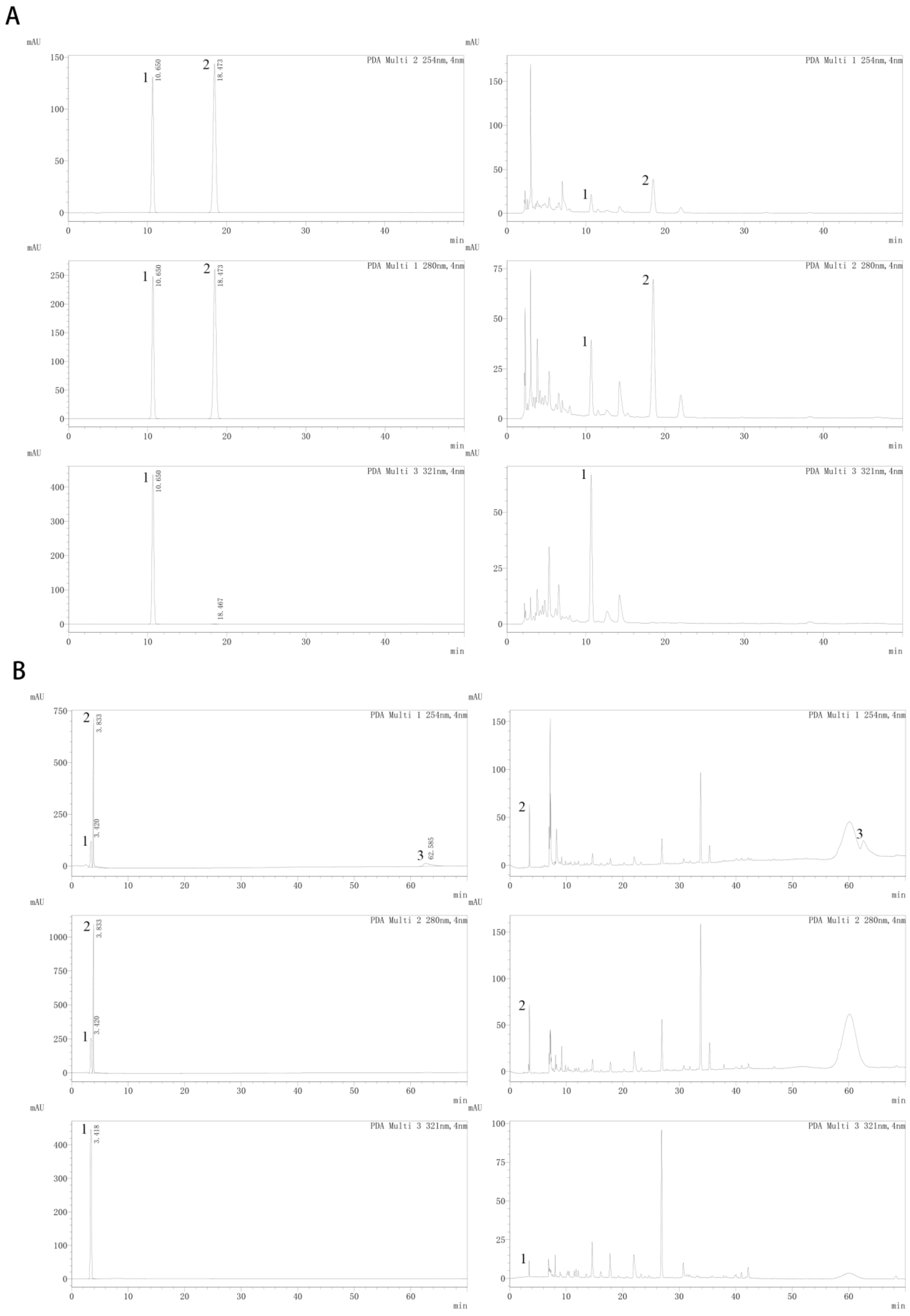

Figure S1. HPLC chromatograms of Chuanxiong Rhizoma aqueous extract

A. Chromatographic separation with WondaSil C_{18} column; B. Chromatographic separation with Shim- pack VP-ODS column. [left, methanol solutions spiked with ferulic acid (peak 1), senkyunolide I (peak 2), and levistilide A (peak 3); right, Chuanxiong Rhizoma aqueous extract].
